# Malfunction defended

**DOI:** 10.1007/s11229-016-1062-8

**Published:** 2016-03-09

**Authors:** Ema Sullivan-Bissett

**Affiliations:** Department of Philosophy, College of Arts and Law, University of Birmingham, ERI Building, Edgbaston, Birmingham, B15 2TT UK

**Keywords:** Generic trait type, Selected functional type, Selected malfunction, Weak etiological malfunction, Selected functions

## Abstract

Historical accounts of biological are thought to have, as a point in their favour, their being able to accommodate malfunction. Recently, this has been brought into doubt by Paul Sheldon Davies’s argument for the claim that both selected malfunction (that of the selected functions account) and weak etiological malfunction (that of the weak etiological account), are impossible. In this paper I suggest that in light of Davies’s objection, historical accounts of biological function need to be adjusted to accommodate malfunction. I propose a historical account which places two conditions on membership of a functional kind. My claim is that it is in virtue of a trait’s meeting these conditions that it is a member of a functional kind, and can thus malfunction. I suggest that a version of my proposal can be adopted by both the selected effects and weak etiological theorists, and so conclude that such a proposal meets Davies’s objection.

## Introduction

Historical accounts^1^ of biological function play an important role in naturalistic theories in the philosophy of mind and language. For example, notions of function are drafted into teleosemantic accounts of mental content (Millikan 1989a; Neander 1995; Papineau 1987). The teleosemantics program provides a naturalistic account of mental representation by appeal to biological function; truth conditions of intentional states are given in terms of what those states are *supposed to do* (Macdonald and Papineau 2006, p. 1). In putting forward an account of mental content which appeals to biological function, one had better have an independent account of that notion. Equally one had better allow for *malfunction *in their account of function, to ground *mis*representation. Historical accounts of function have been thought able to do this important work, and are the choice accounts for philosophers seeking naturalistic theories in the philosophy of mind and language.

However, in several places, Davies launches a systematic attack on the historical account of function, calling it a ‘failed theory’, which ‘does not have the virtues that advocates claim for it’ (Davies 2001, p. 32), and has ‘rather little [...] worth preserving’ (Davies 2001, p. 39). More specifically, he objects to approaches which seek to account for malfunction, and suggests that as naturalists, we ought to do away with the notion of malfunction altogether. Here I address the argument against the possibility of historical malfunction, as presented in Davies’s paper ‘Malfunctions’ (2000) and his book *Norms of Nature *(2001). Davies’s argument teaches us something important about how historical accounts need to be formulated if they are to avoid the ‘internal failing’ of being unable to accommodate malfunction (2001, p. 192). He makes a plea for ‘an argument for the oft-repeated assertion that selected or etiological malfunctions are possible’ (2000, p. 21). I give such an argument (§5). I am not pursuing the more ambitious project of giving an account of malfunction which Davies would accept, since he does not think there is any such phenomenon to give an account of. Rather, I seek to show that an historical approach need not suffer from an *internal failing* to account for malfunction. I am not addressing Davies’s wider aim which is to take norms out of nature altogether, thus eschewing the notion of malfunction simpliciter. I am addressing the objection that the failure to account for malfunction is an internal failing of historical accounts.

As well as offering a revised historical account to meet Davies’s objection, I make two additional and related points. First, I argue that the scope of Davies’s objection is limited. Davies is wrong to claim that historical accounts cannot accommodate malfunction, it is only malfunction due to congenital factors which is problematic (§4). Second, I identify an inherent and under explored commitment in the work of (at least some) historical theorists (§5.1). It is by bringing this commitment to the fore, and taking a lesson from Davies’s objection, that I offer a historical theory of function which possesses the internal resources required for giving an account of malfunction.

Before getting into the topic of the paper, it is worth reflecting briefly on the appropriate methodology for a philosophical analysis of biological function. Two projects should be distinguished, which are distinct, and may be pursued independently. Each brings different adequacy conditions on accounts of function—an account of function may in principle be adequate for one project whilst being inadequate for the other. This does not call either project or account into question, but rather shows that different accounts of function are needed to subserve different theoretical goals.

The first project is driven by philosophy of biology, and the attempt to understand actual cases of biological research. Such an account will be adequate if and only if it is genuinely illuminative of actual cases in biology. The second project is driven by broadly naturalistic considerations within philosophy of mind and language. An account of function designed to serve this project is a theoretical one, adequate if and only if it fulfils the needs of philosophical accounts in these areas. Since, for example, our best naturalistic accounts of mental representation appeal to biological function, and since these accounts appeal to historical functions, Davies’s attack on historical accounts constitutes a real threat to some naturalistic projects in philosophy.

In an incisive paper on the debate over the correct characterisation of biological function, Arno Wouters notes that the recent revival of the philosophical debate on function can be put down to an interest in developing naturalistic theories like these, and not an interest in biology or biological practice (Wouters 2005, p. 123). Philosophers engaged in such projects differ on how they view their accounts of function with respect to whether they are—or should be—in line with actual biological practice. Karen Neander takes her account to be in step with actual biological practice, defending it as conceptual analysis (Neander 1991a). Ruth Millikan on the other hand is clear that she is not engaging in conceptual analysis (1984, p. 18; 1989b, p. 290). Reflecting on earlier work in which she first laid out her account of function, she notes that the point of her notion of *proper function* ‘was/is mainly to gather together certain phenomena under a heading or category that can be used in the construction of various explanatory theories’ (Millikan 1989b, p. 289). She intends her notion of function to be a theoretical definition, one which is able to ‘describe a unitary phenomenon that lies behind all the various sorts of cases in which we ascribe purposes or functions to things’ (Millikan 1989b, p. 293). The idea is to offer a theoretical notion of biological function, and then we can decide if it earns its keep by seeing how much theoretical work it can do.

Participants in the function debate then take themselves to be up to rather different things. If one is interested in how best to understand function attribution in biology, I agree with Wouters that one ought to focus one’s attention on the ‘actual practice of biological inquiry’ (2005, p. 123). However, if one is interested rather in a program like Millikan’s, then one has no such obligation, though one is advised that getting clear on the correct notion of function in this sense will provide ‘little insight in real biology’ (Wouters 2005, p. 123). I am engaging in the second kind of program, and so those philosophers of biology who think that the philosophical notion of function should be in line with actual biological practice may not find much of interest in this paper. However, for those philosophers who are interested in biological function as an explanatory notion which can play a central role in naturalistic theories in the philosophy of mind and language, the work here can be taken as developing a notion of biological function in this sense. Although I think Davies is right that the best extant historical accounts of biological function cannot accommodate all cases of malfunction, I provide a historical account which can.

## The normativity of function and the historical account

Much of the biological function debate has been concerned with accounting for the supposed normativity of functionally characterized items or function statements. An account of function’s ‘main task [...] is to explain how this norm arises in biological contexts’ (Wouters 2005, p. 125), this seems indeed to be the ‘fault line running through [the function] debate’ (McLaughlin 2001, p. 4). Often what the function theorist’s burden comes down to is the requirement to accommodate *mal*function. Malfunctions are taken to be ‘ubiquitous’ (Davies 2000, p. 20). Millikan claimed of function categories that it is an ‘obvious fact’ that ‘their members can always be defective—diseased, malformed, injured, broken, dysfunction, etc.,—hence unable to perform the very functions by which they get their names’ (Millikan 1989b, p. 295).^2^ Carolyn Price claims that it cannot be inferred ‘from the fact that an item has a certain function that it will tend to perform that function: the device may be defective, or the environment in which it operates may not cooperate’ (Price 2001, p. 13).

How can we understand the thought that there is something that biological items are *supposed to do*, that biological items can fail to do what they are *supposed to do*, that they can *mal*function?

Historical accounts look promising. Indeed, it is a ‘persistent boast’ (Davies 2000, p. 19) of this approach, or ‘perhaps the grandest’ of its claims, that it can provide ‘a clear and compelling naturalistic explanation for the occurrence of malfunctions’ (Davies 2001, p. 190). Furthermore, it is claimed by proponents that *only* a historical account can do this work, Millikan says that this is because non-historical accounts ‘run afoul exactly when they confront the most central issue of all, namely, the problem of what failure of purpose and defectiveness are’ (Millikan 1989b, p. 299). She claims that ‘a crippling defect of any definition that looks for function in current dispositions rather than history is that such definitions cannot ground the notion of malfunction’ (Millikan 1993a, p. 32). This is a serious flaw for non-historical theories since ‘[f]unction categories are *essentially *categories of things that need not fulfil their functions in order to have them’ (Millikan 1989b, p. 296).

Proponents of historical accounts claim that a certain historical connection to prior tokens of a trait is necessary and sufficient for a trait’s being a member of a functional kind. This connection sets the norms on proper functional performance, that is, ‘being preceded by the right kind of history is sufficient to set the norms that determine purposiveness’ (Millikan 1989b, p. 299). Biological traits have functions ‘whether or not [they are themselves] capable of serving any of these functions’ (Millikan 1993b, p. 56). A trait can make no causal contribution to fitness, but it nevertheless still has something that it is supposed to do (Neander 1991b, p. 459, fn. 7).

Davies claims that from a naturalistic perspective, it is difficult to see ‘how or why we should think that the processes that result in trait preservation confer upon descendent tokens a norm of performance’ (Davies 2001, p. 67, see also 2009, pp. 139–42). I will give an historical account which has the internal resources to lay down this standard, from which token traits can deviate.

## The supposed impossibility of selected malfunction

Davies’s argument for the claim that historical malfunctions are impossible is, in summary:

If functional types are defined in terms of historical success, then tokens that lack the defining property due to defect, and tokens that have lost the defining property due to disease or damage, are excluded from the functional category. Historically based malfunctions, in consequence, are impossible. (Davies 2000, p. 19)

I will look first at Davies’s objection as applied to the selected functions account as this is its primary target; the argument is applied to the weak etiological account only derivatively.

Davies considers the following account of selected functions. He claims that selected function theorists are committed to these conditions, or equivalents of them. For organism O and trait T, in selective environment E, the function in E of T in O is to do F if and only if:

(i) Past instances of T in O performed F in E,
(ii) T was heritable,
(iii) Past performances of F caused an increase in O’s relative ability to satisfy demands of E (relative to other organisms in the population lacking T),
(iv) This increase in O’s ability to satisfy selective demands of E resulted in an increase in O’s long-term relative rate of reproduction,
(v) This increase in relative reproduction resulted in the persistence or proliferation of O and hence tokens of T. (Davies 2000, p. 22)

To prevent the ascription of functions to vestigial traits, Davies revises the characterization of the selected functions account so that conditions on a trait’s having a function appeal to ‘genealogical descent from a *recently *successful lineage’ (Davies 2000, p. 25, my emphasis). Davies then characterizes the selected functions account thus:

A token of trait T has selected function F if and only if the token is descended from a lineage perpetuated by the recent selective efficacy with which ancestral members performed F. (Davies 2000, p. 25)

Conditions (i)–(v) refer to *recently *successful ancestral tokens of T. Finally, Davies stresses that ‘conditions under which a token trait has a selected function definitely *do not* include possession of the capacity required to actually perform the functional task’ (Davies 2000, p. 25). This is of course granted by, and indeed is one of the insights of, historical accounts.

Davies distinguishes *generic trait types* from *selected functional types*. The former includes all traits which are apt for selection. The latter includes only those variants of a given generic trait type which are selectively successful, that is, those which possess the property selected for. In the oft-cited case of Bernard Kettlewell’s (1973) peppered moth^3^ for example, the targeted trait is wing colouration, and this is the generic trait type. The successful variant of this type is a particular wing colour, and it is this which is the selected functional type.

What kind of trait type does clause (v) concern? This is important: if tokens of T in (v) are members of the generic trait type only, then the selected functions account can accommodate malfunction, because the generic trait type will include instances of the trait which are not selectively successful. So, tokens of T cited in (v) could refer to successful tokens, or unsuccessful (i.e., damaged or diseased) tokens. And the latter, which would qualify as *malfunctioning* tokens, are nevertheless still part of the functional category. This would be a good result for the selected functions theorist.

However, Davies claims that because tokens of T in (i)–(iv) refer to those tokens which possessed the properties which were selected for and are thus members of both the generic trait type and the selected functional type, so too—on pain of equivocation—must be tokens of T in (v). Tokens of T in (v) must refer not just to members of the generic trait type, but to members of the narrower selected functional type. This means that ‘membership in a selected functional category, per condition (v), requires possession of the property selected for’ (Davies 2000, p. 28). It follows that current incapacitated tokens do not belong to the relevant functional types because they lack the capacity which defines that type, despite being descended from a recently successful lineage.

As a result of this, the selected effects account needs to be revised thus:

A token of trait T has selected function F if and only if (1) the token is descended from a lineage perpetuated by the recent selective efficacy with which ancestral members performed F, and (2) the token possesses the property selected for in terms of which the relevant functional category is defined. (Davies 2000, p. 30)

On this formulation, the selected functions account cannot accommodate malfunction. This is because if ‘due to some sort of defect, disease, or damage the token of trait T lacks the property selected for, then it is merely a member of the generic type, not the functional type’ (Davies 2000, p. 30). If this is the case, norms of functional performance do not apply to such tokens. Davies concludes that selected malfunctions are impossible.

We can think of Davies as offering a dilemma. On the first horn, if tokens of T in (i)–(v) refer to members of the generic trait type, then the selected functions account is extensionally incorrect. On the second horn, if tokens of T in (i)–(v) refer to members of the selected functional type, then the account cannot allow for malfunction. Either way, the account is inadequate. I will argue later that this is a false dilemma, since there is another notion of type, more inclusive than selected functional type, but less inclusive than generic trait type. Once we appeal to this more inclusive type, the selected functions account can accommodate malfunction without being extensionally incorrect, since membership of this type is compatible with a trait lacking the capacity to perform the relevant function (§5). Before then, it is worth noting that the scope of Davies’s objection is limited, as I will now show.

## A note on the scope of the objection

Despite Davies’s view that his objection undermines all historical attributions of malfunction, the scope of his objection is much narrower. Even the unmodified selected functions account can in fact accommodate certain kinds of malfunction. Only when traits are unable to perform their function as a result of congenital factors does a problem arise.^4^ This is because conditions (i)–(v) refer to the very existence of a given token, and, as we have seen, a trait belongs to a given selected functional type only if it has the success property for being able to perform its proper function.

However, the set of congenitally damaged token traits is not co-extensive with the set of malfunctioning token traits. Malfunction does not require congenital disease or defect.^5^ Davies claims that ‘it is difficult to see what in the theory of selected functions or in the theory of weak etiological functions justifies the claim that functions persist even when the physical capacity is lost’ (Davies 2000, p. 20). Also, as we saw in his summary of the objection, tokens can ‘lack the defining property due to defect’ and they can ‘[lose] the defining property due to disease or damage’ (Davies 2000, p. 19). Here we see that Davies took his objection to apply to traits which malfunctioned not as a result of congenital disease or damage. Indeed, he claims that ‘an incapacitated token of T is *no longer* a member of the category of Ts’ (Davies 2001, p. 199, n. 6, my emphasis). Even if congenitally damaged traits are not malfunctioning ones because they are excluded from the selected functional category, it does not follow that environmentally damaged traits are excluded from the selected functional category too. It follows only that a necessary condition on function possession is that the trait in question *started* with the capacity to perform the function. To claim necessity for continued possession of that capacity is too strong, or at least, it is not something we can arrive at from Davies’s argument.

The selected functions account can accommodate token traits which *had* the success property, and *were* able to perform their function, but now, due to damage or disease, are unable to do so^6^ (I will call these cases of *environmental *malfunction, as opposed to *congenital *malfunction). These token traits meet the condition put in place by the selected functions account—they hold the appropriate historical connection with previous reproductively successful token traits. Additionally, these token traits meet the condition put in place by Davies—they possess*ed* the capacity to perform their function. They thus count as members of the selected functional type. If they *subsequently* fail perform their function because, for example, they lose the property which granted them membership into the selected functional type, then they malfunction. This means that traits which start as members of the selected functional type but later are unable to perform their function, count as malfunctioning. These will be items such as hearts which *become* unable to pump blood or pancreases which *become* unable to produce sufficient insulin.

Here is a worry with my argument: to say that tokens which had the success property but then lose it due to damage or disease, malfunction, is simply an un-argued denial of Davies’s challenge. For Davies, membership of a selected functional type requires possession of the success property for that type. Just as tokens which do not have that property from the off do not qualify as members of the selected functional type, so too do tokens which *had *the property and then subsequently lose it not qualify as members of the selected functional type. My response to Davies succeeds only because I assume a less strict condition on selected functional type membership than Davies would accept (namely, that a trait merely *start *with the success property).^7^

This objection fails. Tokens of ‘T’ in the selected functions theorist’s conditions on function possession had better refer—on pain of equivocation—to the same kind of thing throughout. And those tokens of ‘T’ had better refer to tokens which actually had the capacity to perform the function, or some success property which enabled the function to be performed. However, Davies adds the further claim that if a token *was *a member of the selected functional type, and *had *the success property, then if it subsequently loses that property (and the capacity to perform the function) due to damage or disease, that token is *no longer *a member of the relevant selected functional type. The selected functions theorist need not accept this. She is forced to accept that tokens of ‘T’ in her five conditions had better refer to tokens with the success property (to avoid equivocation), but she need not accept that those tokens need to *retain* the success property to remain part of the relevant type. This is Davies’s position, but it is not one the selected functions theorist is forced into, and it is not something Davies argues for. Absent positive argument for the more restrictive account, selected functions theorists are free to reject this additional claim. Continued possession of the success property is not something which is required for conditions (i)–(v) to be coherent, and of course, it is something historical theorists are wont to deny.

However, if the selected functions theorist has only this to say, her victory is Pyrrhic. Congenital malfunction is an important kind of malfunction, which a fully adequate account should accommodate. When selected functions theorists discuss malfunction, they often cite examples which are plausibly—at least in some cases—congenital. Millikan talks of members of functional categories which are ‘diseased, malformed, injured, [and] broken’ (Millikan 1989b, p. 295), and Neander talks of items exhibiting ‘pathological deviations from the norm, due to disease, injury or deformity’ (Neander 1991b, p. 467). Millikan and Neander (among others) thus appear to be seeking an account which can accommodate both environmental malfunction *and *congenital malfunction (there is certainly no evidence to suggest otherwise). Further, it could well be that environmental malfunction is not sufficient for the kind of project that, for example, teleosemanticists are engaged in. Davies’s objection therefore remains. A more robust response is required.

## A new historical account of function

Here I give a historical account which can accommodate malfunction (including congenital malfunction). Davies argues that the tokens of ‘T’ cited in (v) have to refer to members of the selected functional type on pain of equivocation, as tokens of ‘T’ cited in earlier conditions refer to members of the selected functional type. However, we do not have to accept that the only referent of ‘T’ is Davies’s proposed *selected functional type, *a type characterised by possession of the success property for functional performance. Instead we can have tokens of ‘T’ in conditions (i)–(iv) referring to members of the selected functional type, and have tokens of ‘T’ in condition (v) referring to members of what I will call the *historical trait type. *What does it take for a token trait to be a member of some historical trait type? I propose placing two necessary and jointly sufficient conditions on function possession and membership of the historic trait type, such that it is possible for the items which meet them to malfunction. To be a member of the historical trait type is to be a member of the functional kind. Membership of a historical trait type (a functional kind), and being ascribable a function, supervene on the same facts (the ones cited in conditions (a) and (b) below).

Evolution does not just target features that construe selective advantages. It targets the underlying heritable physical configuration responsible for possession of the survival-enhancing feature. It is that physical configuration which is primarily heritable hence selectable; the survival enhancing feature is only heritable derivatively, by virtue of heritability of the physical feature that gives rise to it. Moreover, there may be more than one such physical state that gives rise to the feature—that is a form of multiple realizability. However, possession of the physical feature need not necessitate possession of the survival-enhancing one. If membership of the kind goes in part with possession of the physical feature, then membership of the kind will then be compatible with lack of survival-enhancing feature (or performance of the function which defines the functional type). My proposal is the following: functional kinds are associated with sets of properties and particular functions. A token trait *t* is a member of the functional kind *K* and has *F*-ing as its selected function if and only if it:

- (*a*) Possesses one of a set *S* of intrinsic structural properties, $\{p^{1}, p^{2}, p^{3}, {\ldots },\}$
- (*b*) Stands in a causal historical relationship to at least one token trait which
  i. possessed one of a set *S* of intrinsic structural properties, $\{p^{1}, p^{2}, p^{3},{\ldots },\}$
  ii. possessed one of a set $S^{*}$ of properties responsible for that token trait *F*-ing, $\{q^{1}, q^{2}, q^{3},{\ldots },\}$
  iii. was selected for *F*-ing.

Where *selection for* requires variation, in line with the selected functions theorist’s claim that functions are attributed to traits on the basis of what they did which ‘accounts for [their] presence in the population, as over against *historical* alternative traits no longer present’ (Millikan 1993a, p. 40). If a token trait meets the conditions set out here, it is a member of a historical trait type (and a functional kind), and can be attributed a function in the usual way, that is, by looking to its history. Note here that I have moved from talking about the *selected functional type, *as is Davies’s preference, to talking of the *historical trait type, *as is mine. In the next two sub-sections I will say more about the details of these conditions. Explication of condition (a) will take up the most space here, since the positing of a disjunctive set of properties as required for function possession is a large part of what is novel about my approach.

### Condition (a)

Condition (a) picks out a class of intrinsic structural properties. What is required is a set which captures those properties which are possessed by all items to which a particular function would be ascribed, but are not possessed by items which would be ascribed a different function (namely, different traits), or no function at all (namely, severely malformed organic *stuff)*. Here is a rough (and simplified) approximation of one way the historical theorist might go in pinning down the nature of this set (note that I offer this as *one *way, there are no doubt numerous ways tied in with the production of functional traits which might delineate the set of properties in (a)).

We could appeal to an appropriate range of gene expression associated with recent ancestral items which performed *F*. By *gene expression, *I mean to refer to the process through which information contained within a gene is used in the creation of a gene product (e.g. a protein). To qualify as a member of a functional kind *K*,  with the function of *F*-ing, a token trait *t* must have been produced by appropriate gene expression, that is, within a range considered a reasonable attempt to produce an item similar to ancestral items which performed *F*. This set of otherwise heterogeneous intrinsic structural properties cited in (a) can be picked out by an appeal to an appropriate range of allowed departures from the genetic development of ancestral items which performed *F*. This is just to say that there is some set of properties for each functional kind, members of that set may be rather different from one another, but they can be identified *as a set* by appeal to an appropriate range of departures from the genetic development of ancestral items which performed *F*. The idea is that the set of intrinsic structural properties cited in (a) delineate a multiply realizable kind. Past instances of this kind are selected for in virtue of their role in the production of items which were able to perform the function of *F*-ing, which is adaptive (they might, for example, possess property $p^{1}$, or $p^{2}$). This is compatible with malfunction because not every way of realizing this kind needs to be in an *F*-ing contributing way (some realizations of the kind might be in virtue of possessing property $p^{3}$, or $p^{4}$. These properties are within a range of allowed departures of gene expression associated with ancestral selectively successful items). Evolution chooses between intrinsic properties, function attribution is licensed when these intrinsic properties start to have relational functional properties, that is, when condition (b) is met.

I should say something about my use of the terms ‘appropriate range’ and ‘reasonable attempt’. These terms are of course metaphorical insofar as natural selection does not *attempt *anything. Talk in these terms is intended to be helpful in capturing those traits which come pretty close to being well functioning members of a given kind, but nevertheless go awry. That is, those traits for which something goes awry (as in the case of Holt-Oram syndrome below), but does not go *awry enough *to disqualify it from the functional kind (as in the case of what might be best described as ‘glob[s] of misplaced organic matter’ (Millikan 1984, p. 25)). For a token trait to qualify as a member of the functional kind *heart*, we can usefully talk in terms of its being produced within a range considered a ‘reasonable attempt’ to produce something of that kind. How wide is this acceptable range? That will depend on what physical features are selected by natural selection, and that is not something we can pronounce on a priori. The thought is that evolution targets underlying physical structures by virtue of their producing certain traits that confer reproductive advantages. More than one structure or set of properties will be associated with the development of each of these traits. Exactly what counts as the acceptable range is determined by that. This vagueness and imprecision in our language for characterising the set *S* associated with kind *K* and function *F* need not reflect any vagueness or imprecision in actual concrete cases. It is just a by-product of needing a single term to cover the proposal’s structure in abstraction from any given case.^8^

Although admittedly somewhat vague in the abstract, its application in the following case study is hopefully sufficiently clear and precise to alleviate doubts arising from that. Here I run through an example of the genetics of the development of the human heart to show my account at work. The development of the human heart is extremely complicated, and something I cannot do justice to in this short foray into the genetics of this process.^9^ In addition, the genetics of the human heart is a relatively young field, and one which is quickly changing (Pierpont et al. 2007, p. 3015). Let us say that the genetic processes involved in the production of a human heart can produce an array of traits which will fall into one of three categories: *working, defective*, and what I will call *useless* (i.e., Millikan’s ‘globs’). Our appeal to the set of intrinsic structural properties demarcates *working* and *defective* traits from *useless* traits, with those in the former two categories meeting condition (a) on membership of the functional kind *heart. *Such categories will be roughly correlated with the expression of genes which fall into the range considered a reasonable attempt to produce an item similar to ancestral items which pumped blood, and will group together working pumps and defective pumps. And so-called useless traits—excluded from the functional kind—will correlate roughly with the expression of genes associated with the production of ancestral items which pumped blood, but which fall outside of the range of gene expression considered a reasonable attempt to produce such an item.

Take an example of a defective heart, one which my account would classify as a *malfunctioning *member of the functional kind *human heart. *The underlying genetic basis for many heart defects is not known, but what has been found is that mutations in those genes encoding core cardiac transcription factors are involved in many forms of congenital heart disease (McCulley and Black 2012, p. 254). In particular, transcription factors NKX2–5, GATA4, and TBX5 have been identified as playing central roles in the development of the heart, and mutations in these genes are involved in congenital heart disease (McCulley and Black 2012, p. 254, see also Pierpont et al. 2007 for analysis of congenital heart defects associated with single gene mutations). For example, in patients with structural malformations of the heart, mutations in NKX2–5 have been found (McCulley and Black 2012, p. 257, see also McElhinney et al. 2003). In cashing out how condition (a) might apply, I will look at the role of the TBX5 gene in Holt-Oram syndrome.

Holt-Oram syndrome, also known as ‘heart-hand syndrome’, occurs in around 0.001 % of the population. It is a condition characterised by deformities in the upper limbs and congenital heart defects (Pierpont et al. 2007, p. 3024). Research on this syndrome has revealed that it is caused by mutations in a single gene: TBX5,^10^ one of the three genes identified above as playing a central role in human heart development (see for example Basson et al. 1997; McDermott et al. 2005; Li et al. 1997). Most of such mutations in this gene prevent the production of the T-box 5 protein which activates genes involved in the normal development of the upper limbs and the heart. Studies on the role of TBX5 in Holt-Oram syndrome have shown that decreased activity of this gene is responsible for septal defects (most commonly in the wall separating the left and right sides of the heart) (Basson et al. 1997, p. 33). I focus on this example for simplicity’s sake, since little genetic heterogeneity is found among patients with Holt-Oram syndrome^11^ (Pierpont et al. 2007, p. 3025). In this syndrome, we have an example of mutations in a single gene causing abnormal hearts. However, in this case we clearly have something fairly close to a functioning heart and fairly far away from a glob of organic matter. To see this consider some of the macro-level consequences of this syndrome. Transcription of TBX5 is Normally required for septal formation. If this gene is defective, this does not completely destroy or eliminate cardiac function, but merely impairs it. The heart is less effective at what is has been designed to do, but it nevertheless continues to function as a heart in broad terms.^12^ Here we have just one gene which goes awry, this is the kind of case I envisage being characterized as within a range considered a reasonable attempt to produce a heart. This means that the defective hearts of patients with Holt-Oram syndrome are malfunctioning hearts, on my account.

Satisfaction of (*a*) is not sufficient for membership of a functional kind—this is in line with the historical theorist’s claim that an item’s history is necessary for function ascription. If possession of a property in set *S* in (a) was sufficient, then, depending on how we pinned down the nature of the set of intrinsic structural properties, we might get function ascription for first traits (those which are not descendants of *F*-ers with one of the properties causally responsible for the trait *F*-ing) and Swamp^13^ traits (those possessed by the spontaneously coalescing creatures of philosophical thought experiments). Condition (a) can be met by first traits and Swamp traits. When we step back and look at all the traits which were associated with certain sets of gene expression, first traits will be included in this set. As for Swamp traits, they too will meet condition (a) understood in this sense (due to Swampman being a ‘physical replica’ of a biological organism (Davidson 1987, 443)). So these traits may meet condition (*a*) (again depending on how we spell out the nature of the set of intrinsic structural properties), but in their failing to meet condition (*b*), they do not qualify as members of a given functional kind. So they do not have functions, and thus they cannot malfunction.

A worry about condition (a) is that the requirement of a structural property may actually be in tension with historical accounts, because proponents hold that *history is sufficient* for function ascription. It is true that proponents of the selected functions and weak etiological accounts have claimed that history is sufficient to determine purposiveness and norms of performance. However, I think that the appeal to a structural property would not be rejected by historical theorists, since the history which is claimed sufficient is a *certain kind of history*. A brief aside is needed here to defend the claim that the requirement of possession of one of a set of intrinsic structural properties (condition (a)) is not in tension with historical accounts.

Millikan speaks of devices that are ‘*similar *to one another—as human hearts are *similar* to one another’, (Millikan 1984, p. 18, my emphasis). In her definition of a higher-order reproductively established family, as well of talking of ‘*similar *items’, Millikan talks of traits being ‘*in some respects like’* Normal members of a given reproductively established family (Millikan 1984, pp. 24–25, my emphasis). She claims that the use of such terms (‘in some respects like’ and ‘approximates in some degree to a Normal explanation’) reflects the vagueness of the question ‘whether a bit of matter should be called “a malformed eye” or merely “a glob of misplaced organic matter on the forehead”’ (Millikan 1984, p. 25).

How might we to understand what ‘similar’ and ‘in some respects like’ means here? We should not cash out ‘similar’ by appeal to morphological properties, or dispositional properties, because in doing so we cast our net too narrowly—we will exclude malfunctioning traits from our functional categories, as well as traits which simply fail to perform their functions in abNormal conditions. What we can do instead though, is adopt my condition (a) on membership of a functional kind. This would cast the net such that working and malfunctioning traits are distinguished from useless traits. The vagueness Millikan refers to is the kind of vagueness which my condition (a) goes some way to resolve. This is what I am up to when I offer condition (a) on membership of a functional kind. This condition will demarcate *working *and *malformed* items of a given trait type, from what I have called *useless *items.

Earlier I suggested that in cashing out condition (a) we might usefully think in terms of reasonable departures from gene expression. Condition (a) demarcates those working and defective traits from useless traits, with such categories being roughly correlated with gene expression which falls into the range considered a reasonable attempt to produce an item similar to ancestral items which performed *F*. This story sit very well with Millikan’s claims, bearing ‘*some resemblance*’ and matching ‘*in some relevant respects*’ can be cashed out in terms of producing an item within a range considered a reasonable departure from the gene expression associated with selectively successful ancestral tokens of the functional kinds.

Turning now to Price, a weak etiological theorist. She too indicates that the requirement of a structural property would not be unwelcome. When discussing Andrew Woodfield’s (1976) account of function statements (what she calls an ‘Actual Capacity Account’ (ACT)), she suggests that the ACT theorist should borrow a criterion of Millikan’s, one which ‘focuses on the causal origin of the device: two devices belong to the same group if they were produced by the same genetic mechanisms in (*more or less*) the same way’ (Price 1995, p. 147, my emphasis). And, in a footnote attached to this claim, Price claims that the ‘phrase “more or less” is needed to allow for malformed devices’, a claim she attributes to Millikan (1984, p. 25) (Price 1995, p. 147, fn. 9). In discussion of her own account, Price claims that ‘two devices will count as belonging to the same type if they were produced by the second system, or its ancestors, in *more or less* the same way’ (Price 1995, p. 151, my emphasis).

In later work, Price claims that ‘[a]n item will be equivalent to one of these earlier items if it is causally related to [some token device] in *much the same way* as those earlier items were related to earlier members of [some type device] that assisted them to [perform some function]’ (Price 2001, p. 38, my emphasis). And in a footnote to this claim she states that the ‘processes that produced my heart and my liver were related to each other in *more or less* the same way as the processes that produced my ancestor’s heart and her liver’ (Price 2001, p. 38, fn. 5, my emphasis).

This ‘in much the same way’ and ‘more or less’ can be understood in my terms. If two token traits were produced within an appropriate range of gene expression associated with recent ancestral items which performed *F*, they are ‘produced by the same genetic mechanisms in (*more or less*) the same way’ (Price 1995, p. 147, my emphasis). They can also both be said to be ‘causally related to [some token device] in *much the same way* as those earlier items were related to earlier members of [some type device]’ (Price 2001, p. 38, my emphasis).

So requiring a structural property for membership of a functional kind would be friendly to both selected functions and weak etiological accounts, and would help pin down more precisely what Millikan^14^ and Price^15^ are getting at when they talk in terms of items being ‘similar’ or produced ‘in more or less the same way’. It is in bringing out and developing this underexplored commitment in the work of some historical theorists that we can formulate a revised historical account of function which can accommodate malfunction. This is achieved by making explicit the requirement of an intrinsic structural property *in the conditions *on functional kind membership.

Getting more specific about the kind of intrinsic structural property required is tricky,^16^ and though I have offered a way historical theorists might go with regards to this, it is inevitable that vagueness will arise. Though vagueness of this kind will infect any account of biological function owing to borderline cases, and so it does not speak against the conditions on function ascription I have offered. This is recognised—indeed embraced—by historical theorists. Millikan claims that there ‘are lots of borderline cases of proper function, if not so many in nature, certainly in possible worlds (Millikan 2002, p. 115, fn. 2). When discussing the development of functional terms (‘Cummins biofunction’ and ‘proper biofunction’) she suggests that we should ‘*not *attempt to give these notions entirely clean boundaries. Nature has many important joints, but these joints are seldom clean’ (Millikan 2002, p. 122, my emphasis). Similarly Price claims that there ‘is nothing wrong with a certain amount of vagueness in our account of functions provided we are clear about what is going on’ (Price 2001, p. 29, fn. 19).

### Condition (b)

Let us now look to (*b*). The three clauses in (b) are each doing important work, they make specific the causal relationship required to hold between a present day token and an ancestral token of a trait type, by requiring that the ancestral token satisfies clauses [i], [ii], and [iii]. Let us first look to (b) [i]. Requiring the ancestral trait in the causal-historical relation to possess one of a set *S* of intrinsic structural properties, $\{p^{1}, p^{2}, p^{3},{\ldots },\}$ rules out the possibility of functional kind membership for traits which are descendants of something which performed *F* but are a different kind of thing, for example, a blood-pumping pseudo-lung (note that I do not call it a *lung* because biological kinds are typed by their functions). The property in (b) [ii] is the success property, the property which allows the function to be performed, if a trait has this property it is a member of Davies’s *selected functional type.* This clause is important because there must be historical success for function attribution. Finally, clause [iii] of (b) is present to reflect the selected functions theorist’s commitment to the claim that variation is required for selection. That is, a trait has the function it does because the historical performance of that function accounts for the trait’s presence in the current population, ‘as over against *historical* alternative traits no longer present’ (Millikan 1993a, p. 40).

Importantly, meeting the three clauses in (b) is not sufficient for function attribution. The historical connection to, for example, ancestral human hearts cannot be enough for functional kind membership for at least two reasons. First, my lungs hold a historical connection to ancestral hearts (since my ancestors had hearts), but that does not mean that my lungs are part of the function kind *heart *and have pumping blood as their proper function. Second, imagine the case in which something goes *so* wrong, that the trait produced is a so-called useless one. The resulting product does not come close to being a heart. These items are not *malfunctioning* items, but rather not functional traits at all. Condition (a), the requirement of an intrinsic structural property, excludes such items from the functional category.

In summing up the presentation of my position, it is important to note how I differ from previous historical accounts of function. My account is very much influenced by Millikan’s, and so I will note the ways in which I depart from her, especially with respect to accommodating malfunction.

In her definitions of higher-order *reproductively established families* Millikan gives a condition on membership to some functional kind *R* as its having ‘been produced in accordance with an explanation that approximates in some (undefined) degree to a Normal explanation for production of members of *R*’ (Millikan 1984, p. 25). She wants to make room for malformed members of *R* by appeal to this similarity condition (though see fn. 2).

In my account, I make room for malfunction by appeal to a range of gene expression associated with ancestral items which were selectively successful. This range captures a set of otherwise heterogenous intrinsic structural properties, which includes *working *and *defective *items, and excludes *useless *items. Introducing the idea of functional kinds being associated with *sets of properties, *allows functional kindhood to be multiply realizable *by appeal to these sets of properties. *Not every way of realizing some functional kind needs to be one which realizes it in virtue of being selectively successful.

Here is a (rather dramatic and admittedly toy) case where my account and Millikan’s license different judgements. Suppose radiation poisoning causes gene expression which produces a heart which cannot perform the function of pumping blood without artificial assistance. If that gene expression were within the range associated with ancestral tokens of the functional kind heart, but the deviation from the ideal were caused by radiation, my account has it that the resulting product would be a *malfunctioning heart. *For Millikan though the resulting product would not be a *malfunctioning* heart, but a heart produced under abNormal conditions.^17^

Millikanian Normal conditions and Normal explanations are indexed to a distinctive historical environment. In contrast, I place the emphasis on gene expression in an environment which need not be a Normal one. My account is thus less relational than Millikan’s. Her talk of items being produced in accordance with an explanation that approximates a Normal explanation, raises the question of whether something could be unable to perform its function in a given environment in a way which could properly be described as *malfunctioning* as opposed to just failure to perform (hence why Millikan is doubtful about putative cases of malfunction, see fn. 2). If one is committed, as Millikan is, to restricting cases of malfunction to ones in which the item is produced in accordance with an explanation which approximates a Normal one, malfunction will be very rare indeed. I am not committed to such a restriction.

### The possibility of selected malfunction

If the selected functions theorist adopts my proposal, she can now meet Davies’s objection by reading ‘tokens of T’ in conditions (i)–(v) as follows:

(i) Past instances of tokens of the *selected functional type* in O performed F in E,
(ii) The *selected functional type* was heritable,
(iii) Past performances of F caused an increase in O’s relative ability to satisfy demands of E (relative to other organisms in the population which do not have tokens of the *selected functional type*),
(iv) This increase in O’s ability to satisfy selective demands of E resulted in an increase in O’s long-term relative rate of reproduction,
(v) This increase in relative reproduction resulted in the persistence or proliferation of O and hence tokens of the *historical trait type*.

Though the referent of ‘T’ changes between conditions (i)–(iv) and condition (v), this is unproblematic. This is because now, the selected functions account has it that the success of members of the selected functional type resulted in tokens of the historical trait type. And tokens of the historical trait type can malfunction, because (a) they possess one of a set of intrinsic structural properties, and (b) they are governed by the norms of performance bestowed on them by their historical connection to prior successful tokens of the generic trait type to which they belong. As long as a trait is part of the historical trait type, then the norms of performance apply to it, and it can malfunction.

To be clear: I conceded that Davies has shown that the referent of tokens of ‘T’ needs to be such that equivocation is avoided. I suggested that equivocation can be avoided by appealing to the *historical trait type, *members of which meet my conditions (a) and (b) on function ascription. My response is to the charge that historical accounts do not have the internal resources to account for malfunction. Davies thinks that the referent of ‘T’ throughout conditions (i)–(v) should be the selected functional type. The historical theorist need not accept this proposal. Rather, the success of members of the selected functional type can result in members of the historical trait type without equivocation. This revised selected functions account can accommodate malfunction.

## The supposed impossibility of weak etiological malfunction

The weak etiological account of biological function differs from the selected functions account in that variation during the period in which a trait contributes to organismic fitness is not a necessary condition on function possession. That is, a trait *T* can have the function to *F* providing ‘*T* contributed to the fitness of the ancestors of *T*’s current bearers *by* producing an effect *E* and that this, in turn, contributed to the reproduction of *T*s’ (Buller 1998, p. 508). The weak etiological account then is more liberal than the selected functions account, which requires variation for function ascription. On the selected functions account, as we have seen, for a trait *t* to have the function of *F*-ing, ‘an item must not just contribute to survival or reproduction, it must contribute to reproduction better than some alternative item, that is, it must contribute to differential reproductive success or fitness’ (McLaughlin 2001, p. 106).

Davies formulates the weak etiological account thus,

A token of trait T has weak etiological function F if and only if (1) ancestors of this token contributed to some component of organismic fitness by performing F and (2) T was heritable. (Davies 2000, p. 33)

Davies suggests that the eschewal of the appeal to selection ‘may give the appearance that [his] argument [...] does not apply to the theory of weak etiological functions’ (Davies 2000, p. 34). However given certain adjustments, the argument applies here also. The adjustment is to cast the argument not in terms of selective success as before, but more generally in terms of properties of historical success (Davies 2000, p. 34).

Even though for the weak etiological theorist variation is not necessary for functional kind membership, for any case, there either was variation among ancestral instances of the trait in question, or there was not variation. Davies argues that whichever is the case, his objection goes through. If there was variation, the weak etiological functional category gets defined by reference to the successful variants that contributed to ancestral fitness by performing *F*. So, as before, if a present day token does not possess the capacity to perform *F* due to defect, disease, or damage, that token is not a member of the functional kind, and so it cannot malfunction. If there was not variation, the objection still goes through. Davies supposes that ‘not a single variant was sufficiently defective, diseased, or damaged to prevent it from contributing to fitness’ (Davies 2000, p. 34). Now the weak etiological functional category gets defined by reference to the tokens that contributed to ancestral fitness by doing *F*, in this case it just so happens to be all ancestral tokens. But again, present day tokens which lack the capacity to perform *F*, do not count as members of the functional type. So weak etiological malfunctions, as well as selected malfunctions, are impossible (Davies 2000, p. 34).

## The possibility of weak etiological malfunction

The weak etiological theorist can respond in much the same way as the selected functions theorist did. There will be a change in the formulation to reflect the difference between the accounts. My proposal for a revised weak etiological account is the following: a token trait *t* is a member of the functional kind *K* and has *F*-ing as its selected function if and only if it:

- (*a*) Possesses one of a set *S* of intrinsic structural properties, $\{p^{1}, p^{2}, p^{3}, {\ldots },\}$
- (*b*) Stands in a causal historical relationship to at least one token trait which,
  i. possessed one of a set *S* of intrinsic structural properties, $\{p^{1}, p^{2}, p^{3},{\ldots },\}$
  ii. possessed one of a set $S^{*}$ of properties responsible for that token trait *F*-ing $\{q^{1}, q^{2},q^{3},{\ldots },\}$
  iii. contributed—by *F*-ing—to some component of organismic fitness.

The only difference between this formulation and the one offered earlier is in condition (b [iii]). The earlier formulation had as (b [iii]) ‘was selected for *F*-ing’, making it a condition on functional kind membership that a trait was descended from a trait which was selected for, where being selected for requires variation among ancestral traits. As we have seen though, the weak etiological account does not require this sense of selection for function attribution, it requires something much weaker, namely, a trait’s having contributed—by *F*-ing—to some component of organismic fitness, and it is this requirement that constitutes clause [iii] in (b).

Again, call the traits which meet conditions (a) and (b) members of the *historical trait type*. Membership of a functional kind is not defined by reference to tokens which contribute to fitness but rather, as with the selected functions account, is defined by reference to traits which met conditions (a) and (b). The weak etiological account now has it that success of members of the selected functional type—those members which contributed to organismic fitness—resulted in tokens which are members of the historical trait type. And, once again, tokens of the historical trait type can malfunction, because (a) they possess one of a set of intrinsic structural properties, and (b) they are governed by norms of performance bestowed on them by their historical connection to prior tokens of the generic trait type to which they belong which contributed to organismic fitness. If a trait is a member of the historical trait type, then it can malfunction in virtue of there being norms of performance which apply to it. This revised weak etiological account can also accommodate malfunction.

## Conclusion

In this paper I have done three things. First, I have shown that the scope of Davies’s objection is limited—some historical malfunctions survive unscathed. Second, I argued that the objection can be answered by my revised historical account. Third, I identified a component of historical theories which have escaped attention. Once that component is brought to the fore, Davies’s challenge can be met by a revised historical account. I proposed a new account according to which there are two conditions on membership of a functional kind. If the historical theorist adopts these conditions, she does not have to accept that functional kinds are defined by reference to successful tokens, it was this which made her account vulnerable to Davies’s objection. Rather she can hold on to the historical component of her account—it is after all by reference to a token trait’s history that a specific function can be specified—and, by adding the requirement of an intrinsic structural property to the conditions on membership of a functional kind, she can distinguish malfunctioning tokens of a given type from so-called useless ones. As I noted at the start of the paper, my aim here has not been to give an account of malfunctions which Davies would accept, since he would not accept any account of malfunction, on the grounds that no account is required. I conclude then that Davies’s challenge can be met by my historical account of biological functions, and that historical malfunctions—of both the selected and weak etiological kind—are possible.

## Footnotes

1 References to historical accounts should be taken to refer to both strong (selected functions) and weak etiological accounts. When referring to those accounts in particular I will use these terms.

2 There is an important distinction between an item *failing to perform its function*, and an item *malfunctioning*. In later work, Millikan is more doubtful about whether anything falls into this second category, and so she thinks that the selected functions account need not concern itself with accounting for malfunctioning items—this work can be done by appeal to abNormal conditions (a ‘Normal condition’ [note the capitalization] is a condition which must be mentioned in a description of the proper performance of an item’s function (Millikan 1984, p. 33). Compare: *sperm Normally fertilize ova*, with *sperm normally fertilize ova*—the first is true, the second is false). So we should not say that an item is malfunctioning, rather we should say that an item is failing to perform its function because conditions are abNormal. Given this story, Millikan thinks that malfunction proper is actually very rare (if indeed it happens at all), and that an item failing to perform its function is the ‘usual case’ (personal correspondence). (See Millikan (1997) for the first statement of this view, and more recently, see Millikan (2013, p. 40).) This will be important later when I discuss the differences between my account and Millikan’s (§5.2). For now I will just note that theorists differ on the extent to which malfunction is possible, and its prevalence. For those who think it is possible, and that it is something which an account of biological function ought to accommodate, I offer an account which can do so. The point here is to show that a historical account can—if necessary—accommodate malfunction.

3 Before 1850, near Manchester, tree trunks roosted on by peppered moths were lichen-covered. This meant that those moths with light coloured wings were protected through camouflage, from predation from birds, whilst moths with dark coloured wings were heavily preyed upon. Then industrial soot destroyed the lichen, resulting in a darkening of the tree trunks. By the end of the nineteenth century, the selective successes of the moths had been reversed. Now the dark coloured winged moths were protected from predation, and the light coloured winged moths were exposed to it. Whereas before 99 % of the peppered moth population had light coloured wings, now 99 % of the peppered moth population had dark coloured wings (see Kettlewell 1955, 1958). Such results were not specific to Manchester, rather there was a ‘correlation between the frequencies of the melanic forms and the industrial areas of Britain’ (Kettlewell 1958, p. 58).

4 There is of course a question about whether all of these kinds of cases will be due to abNormal conditions, rather than malfunction (see fn. 2). I suspect Neander would think that these cases are cases of malfunction, whereas Millikan would say that they are cases of mere failure to perform, due to conditions being abNormal for proper functional performance.

5 See also Maarten Franssen’s argument against Davies. Franssen takes issue with Davies’s reading of conditions (i), (iii), and (iv) of the selected effects account, on the grounds that Davies reads them as applying to *every *token of T (i), or *every *organism (iii) and (iv). He suggests, following Neander (1991a, p. 174) that the ‘causal explanation of the selection does not require the positive contribution of every historical possessor of tokens of the type corresponding to the selection of the genotype’ (Franssen 2009, p. 108). This means that we can appeal to the *average *contribution of tokens of T, and so tokens of T in (v) can be malfunctioning ones.

6 This ‘due to damage or disease’ qualifier is crucial. It is not my position that every case of loss of function-relevant capacities is a case of malfunction (consider non-infant humans’ loss of the instinct to suckle, this is not a case of a suckling malfunction). We can appeal to Normal conditions to principally distinguish function loss as *malfunction *from function loss as failure to perform a function in abNormal conditions. The conditions in which a non-infant human finds itself are conditions which are abNormal for the performance of the suckling function, and so this loss of function-relevant capacities is not a case of malfunction. I am grateful to an anonymous referee for seeking clarification on this point.

7 I am grateful to an anonymous referee for raising this objection.

8 I am grateful to two anonymous referees for encouraging an explanation of my language here, and to Nicholas Jones for helpful discussion.

9 See Marc Sylva et al. (2014) for an excellent step-by-step description of human heart development, especially helpful to the uninitiated!

10 The full(er) story: The TBX5 gene encodes T-box 5 protein, a transcription factor which goes on to regulate the expression of other genes (and so proteins produced by other genes). A mutated TBX5 gene may produce a changed protein, which cannot activate a subset of genes required for heart development, which results in heart defect. This changed protein though may be able to activate genes required for lung development, so mutations in the TBX5 gene cannot function in heart development, but still function fine elsewhere. Thanks to Chelsea Brain for discussion on this.

11 Rarely can we isolate a single gene as responsible for a congenital heart defect, and so I note here that much more complicated stories would need to be told if one were interested in how my account of function would characterize human hearts with different defects, since human heart defects ‘are an etiologically heterogeneous collection of malformations, with overlapping genetic and environmental factors that contribute to disease presentation’ (Moran and Robin 2013, p. 169). The genetic homogeneity of this syndrome makes it an ideal one for my purposes, since it allows for a relatively simple explication of condition (a), whilst still referring to a real example.

12 Thanks to Simon Barnes for discussion on this.

13 The Swampman thought experiment comes from Davidson (1987). Imagine that I, and a dead tree in a swamp, get struck by lightning. My body is ‘reduced to its elements’, whilst the tree is turned into a molecule for molecule identical version of me: Swamp Ema. If one is a historical theorist about function, Swamp Ema’s blood-pumping organ is not a member of the functional kind *heart* and does not have the function of pumping blood. This is because spontaneous coalescence is not the appropriate history for function ascription.

14 Millikan does not think that malformed devices are terribly important to the historical theorist’s program (see fn. 2), though she thinks we might account for a malformed device using the conditions I introduce (personal correspondence).

15 Price agrees that appealing only to history will not do, and that we need another (merely necessary, not sufficient) condition to meet Davies’s objection, though we should prioritize the historical condition (personal correspondence). This is in line with what I have done. Condition (a) allows us to meet Davies’s objection, whilst condition (b) is still necessary (and allows us to specify the function of a given trait type). Price also she thinks that we ought to prioritize similarity of *developmental process* over similarity of *resulting product*. I suspect those similarities might be roughly co-extensive—I doubt we would get a useless glob if something were produced in a similar enough way to the way ancestral hearts were produced, equally, I doubt we would get a heart, if the way in which it was produced was not similar to the way in which hearts were produced. So, if one sympathises with Price on this point, I think condition (a) could be cashed out in these terms; instead of requiring the possession of one of a set of intrinsic structural properties, we might instead require that the item be produced within a range of ways considered a reasonable attempt at producing an item which could perform *F*.

16 I am not seeking to give a definitive pinning down of condition (a) here, but only to argue that something like condition (a) is necessary. I offer one way in which this might be done above.

17 Thank you to Paul Noordhof for suggesting this example as illuminative of one of the differences between my account and Millikan’s.
